# 3D Ultrasound and MRI in Assessing Resection Margins during Tongue Cancer Surgery: A Research Protocol for a Clinical Diagnostic Accuracy Study

**DOI:** 10.3390/jimaging9090174

**Published:** 2023-08-28

**Authors:** Fatemeh Makouei, Tina Klitmøller Agander, Caroline Ewertsen, Morten Bo Søndergaard Svendsen, Rikke Norling, Mikkel Kaltoft, Adam Espe Hansen, Jacob Høygaard Rasmussen, Irene Wessel, Tobias Todsen

**Affiliations:** 1 Department of Otorhinolaryngology, Head and Neck Surgery and Audiology, Rigshospitalet, Copenhagen University Hospital, DK-2100 Copenhagen, Denmark; 2 Institute of Clinical Medicine, Faculty of Health and Medical Sciences, University of Copenhagen, Blegdamsvej 3, DK-2200 Copenhagen, Denmark; 3 Department of Pathology, Rigshospitalet, Copenhagen University Hospital, DK-2100 Copenhagen, Denmark; 4 Department of Radiology, Rigshospitalet, Copenhagen University Hospital, DK-2100 Copenhagen, Denmark; 5 Copenhagen Academy for Medical Education and Simulation, The Capital Region of Denmark, DK-2100 Copenhagen, Denmark; 6 Department of Computer Science, University of Copenhagen, 2100 Copenhagen, Denmark

**Keywords:** 3D ultrasound imaging, surgical margin assessment, magnetic resonance imaging, tongue squamous cell carcinoma, ex vivo surgical specimen imaging, squamous cell carcinoma histopathology, oral cancer

## Abstract

Surgery is the primary treatment for tongue cancer. The goal is a complete resection of the tumor with an adequate margin of healthy tissue around the tumor.Inadequate margins lead to a high risk of local cancer recurrence and the need for adjuvant therapies. Ex vivo imaging of the resected surgical specimen has been suggested for margin assessment and improved surgical results. Therefore, we have developed a novel three-dimensional (3D) ultrasound imaging technique to improve the assessment of resection margins during surgery. In this research protocol, we describe a study comparing the accuracy of 3D ultrasound, magnetic resonance imaging (MRI), and clinical examination of the surgical specimen to assess the resection margins during cancer surgery. Tumor segmentation and margin measurement will be performed using 3D ultrasound and MRI of the ex vivo specimen. We will determine the accuracy of each method by comparing the margin measurements and the proportion of correctly classified margins (positive, close, and free) obtained by each technique with respect to the gold standard histopathology.

## 1. Introduction

Oral squamous cell carcinoma (SCC) is the eighth most common cancer worldwide, with less than 60% of the patients surviving more than 5 years [1]. In a study conducted at our center (Department of Otorhinolaryngology, Head and Neck Surgery, and Audiology, Rigshospitalet, University of Copenhagen, Copenhagen, Denmark), among 8299 oral cancer patients, it was found that the 5-year overall survival rate was 43% for women and 36% for men [2]. SCC of the tongue is the most common type of oral cavity cancer, and the primary treatment for patients with early stages of tongue SCC is surgery [3,4]. The main goal of surgical oncology is to entirely remove the tumor, along with a margin of healthy tissue surrounding it, to ensure proper cancer removal. At the same time, preserving as much healthy tissue as possible during tongue cancer surgery ensures the quality of life for the patient, maintaining vital functions such as swallowing and speaking after surgical treatment. Survival and the need for postoperative treatment are highly dependent on the accuracy of the surgical resection margins for the cancer. Inadequate surgical margins (margin ≤ 5 mm) may lead to a second resection [5]. The presence of positive (<1 mm) or close (1–5 mm) margins following cancer surgery leads to an increased risk of local cancer recurrence, reduces the chance of survival, and necessitates adjuvant treatments, such as radiotherapy or chemoradiation. In a study at our center investigating 1399 patients with oral squamous cell carcinoma (OSCC), 44% of the cases received adjuvant radiotherapy after surgery [6]. A recent retrospective analysis of 91 tongue SCC resections reported a total of 74% of the cases with close margins (1–5 mm) and 10% with positive margins (<1 mm) [7]. Given the significant impact of surgical precision on patient outcomes in oral squamous cell carcinoma (SCC), there is a pressing need for advanced intraoperative techniques to ensure complete tumor resection. The potential for positive surgical margins, which can lead to the necessity for additional treatments or even further surgery, underscores the critical need for more accurate and efficient intraoperative techniques. Therefore, the development and implementation of innovative approaches that can improve the rate of complete tumor resection are not only desirable but essential for enhancing surgical care and ultimately improving patient survival rates and quality of life [8].

Frozen section biopsy is routinely used for intraoperative margin evaluation in carcinomas of the head and neck. During this procedure, a separate tissue fragment representative of each margin is excised from the operation field after resection of the primary tumor and individually submitted as frozen sections to be microscopically evaluated by the pathologist. One of the main limitations of frozen section analysis is that only a few margins (the ones which are nearest to the tumor) can be examined during surgery [9]. Furthermore, this procedure increases the time in anesthesia by 30–60 min, increasing the risk of patient complications, as well as the treatment cost.

Another technique is the assessment of surgical margins by fluorescence imaging [10]. There are some challenges with this technique, such as the inter-/intra-variability of antibody agent concentrations between patients and within tumors [10]. However, the main challenge regarding the use of fluorescence imaging techniques is their limitation to the resection surface, as they do not provide information from the deep tissues inside the specimen. 

Perioperative or intraoperative MRI of surgical specimens has been suggested to improve the evaluation of the resection margins during cancer surgery. Heidkamp et al. implemented an intraoperative method to scan tongue cancer specimens but did not find it accurate in assessing resection margins [11]. In contrast, another study found MRI to reliably predict close or positive resection margins following tongue cancer surgery [12]. Still, MRI is a costly imaging modality, and other more portable imaging techniques may be preferable in the operating room.

Ultrasound is a portable, dynamic, cost-efficient imaging modality that can be used to provide high-resolution visualization of surgical specimens [13]. Often, 2D ultrasound has been used to visualize and measure in vivo tongue tumors [14,15,16]. A better prediction of tongue carcinoma staging is obtained using ultrasound compared to the use of manual palpation [14]. The depth of invasion (DOI) is an important factor for the T-classification of tongue SCC. Nilsson et al. assessed DOI in 40 patients with T1–T3 tongue SCC using 2D ultrasound, palpation, computed tomography (CT), and MRI, and it was concluded that 2D ultrasound was the most accurate method of evaluation [4]. A systematic review investigated the feasibility of 2D ultrasonography to measure the tumor thickness of oral cancer. It was concluded that there was a high correlation (r = 0.82, *p* < 0.001) between ultrasound and histopathology in tumor thickness measurement [15]. The result of another review article has shown the promising correlation between ultrasound and histopathology in tongue SCC for cervical lymph node metastases [16]. In another study, it has been argued that the tumor thickness measurements obtained by ultrasound can provide information about the cellular dissociation grading of tumor budding and cell nest size in early tongue cancer [17]. 

Moreover, 2D ultrasound has also been used for ex vivo imaging of tongue tumors [7,18,19]. De Koning et al. performed ex vivo 2D ultrasound examination of the margins in resected tongue SCC, which resulted in reduced need for local adjuvant treatment [7], and in another study, they showed that ultrasound-guided ex vivo tongue SCC resections improved the margin status and reduced the frequency of adjuvant radiotherapy by 50% [18]. Adriaanses et al. measured the tumor thickness using ultrasound and MRI and compared the results to those obtained by histology in 13 buccal mucosa SCC [19]. The accuracy of in vivo and ex vivo ultrasound for measuring tumor thickness was comparable to that of MRI. 

The limitations of most of the studies with 2D ultrasound imaging include the user-dependency and generation of dynamic two-dimensional image “slices” of the tissue using ultrasound. Instead, 3D ultrasound imaging is a promising approach to obtaining volumetric ultrasound images of the tumor and overcoming some of the problems of conventional B-mode ultrasound. The main advantages of 3D ultrasound compared to conventional 2D ultrasound imaging include the 3D visualization of the entire structure, less operator dependency, orientation-independent visualization, the measurement of quantitative attributes in three dimensions, the repeatability of the region of interest (ROI) examination, and the possibility of fusion with cross-sectional imaging modalities [20]. A list of research works, including some of the different 3D ultrasound imaging techniques and their applications, has been summarized in Table 1. 

There are studies reporting a higher diagnostic accuracy using 3D ultrasound compared to conventional 2D B-mode imaging [34,35]. We also conducted a pilot animal study to compare 3D ultrasound with CT, without a contrast agent, in measuring plane-by-plane tumor areas in an animal model. The result was a higher correlation between 3D ultrasound and the gold standard gross pathology [30]. In the head and neck region, freehand 3D ultrasound has previously been used for 3D imaging of jugular vessels [36,37], cervical lymph node assessment [38], and improving the tumor resection in glioma surgery [39].

Compared to MRI and CT, 3D ultrasound has the advantage that it can be performed immediately in the operating room in less than 10 min. It can also make an evaluation of the entire resected specimen, instead of the 0.1–1% samples sent for frozen section analysis [18,40].

Bekedam et al. used electromagnetically tracked ultrasound imaging to scan eight tongue cancer specimens immediately after excision [21]. The specimen and the tumor were segmented manually by a radiologist, and the margins were computed in 3D by calculating the closest Euclidean distances between the segmented specimen and the tumor boundary. The borders were more difficult to distinguish in some of the specimens due to small echogenicity differences between the tumor and healthy tissue. Even though the use of electromagnetic sensors is one of the most cost-effective methods for localization in freehand 3D ultrasound systems, their main drawback is their metal sensitivity [20]. Table 2 summarizes some of the publications focusing on surgical margin assessment.

In the current protocol, we introduce a novel approach to the assessment of resection margins in tongue SCC. We propose the application of 3D ex vivo ultrasound using a mechanical arm and a custom-made setup (3Sonics). This technique represents a significant advancement in the field, as it offers a portable, cost-effective, and efficient alternative to existing 3D ultrasound imaging methods. Unlike other imaging techniques, our proposed 3D ultrasound imaging technique does not rely on any type of sensor for probe position tracking, simplifying its operation and potentially increasing its accessibility and usability in various clinical settings. 

Furthermore, our method allows for a comprehensive comparison of the 3D ultrasound outcomes with results from MRI and clinical examination of the surgical specimen by a surgeon. This multi-modal approach enhances the robustness of our study and provides a more comprehensive understanding of the resection margins. The diagnostic accuracy of our method will be evaluated by computing the correlation of these three approaches to the final histopathology report, the gold standard in cancer.

## 2. Research Question

In patients surgically treated for oral tongue squamous cell carcinoma, what is the accuracy of 3D ultrasound compared to clinical examinations and MRI in the assessment of tumor resection margins?

## 3. Materials and Methods

The diagnostic accuracy study will be conducted at the Department of Otorhinolaryngology, Head and Neck Surgery, and Audiology, University Hospital of Copenhagen, Rigshospitalet (RH), Copenhagen, Denmark. The protocol adheres to recommendations for interventional trials described in the SPIRIT and STARD guidelines [47]. 

Patients diagnosed with tongue SCC will be invited to participate in the study. All participants meeting the inclusion criteria (described in Section 3.1) will undergo a preoperative in vivo ultrasound scan to measure the depth of invasion. Perioperative ex vivo 3D ultrasonography of the resected tongue tumor will then be performed. Furthermore, the surgeon will assess the surgical margins by clinical examination of the resected specimen ex vivo. Then, the surgical specimen will be MRI scanned at Department of Radiology immediately after surgery. Afterwards, the surgical specimen will be transferred to the pathology department for a formalin fixation procedure. On the day after the surgery, the surgical specimen will also be scanned by 3D ultrasound after formalin fixation. Surgical margin assessment results from the surgeon assessment, MRI, and the 3D ultrasound scan of the surgical specimen will be correlated to the final histopathology report. 

### 3.1. Eligibility Criteria

We will invite patients with tongue cancer scheduled to undergo surgical treatment at Rigshospitalet. The department performs all oncological surgical treatment of head and neck cancer for a population of about 2.6 million people from the Eastern Region of Denmark (comprising 46% of the Danish population), as well as Greenland and the Faroe Islands. Patients who meet the inclusion criteria (see Table 3) and agree to participate in the study will be enrolled in the trial.

### 3.2. Perioperative Assessment of Margins 

#### 3.2.1. Surgical Specimen Examination by the Surgeon

**Figure 1:**
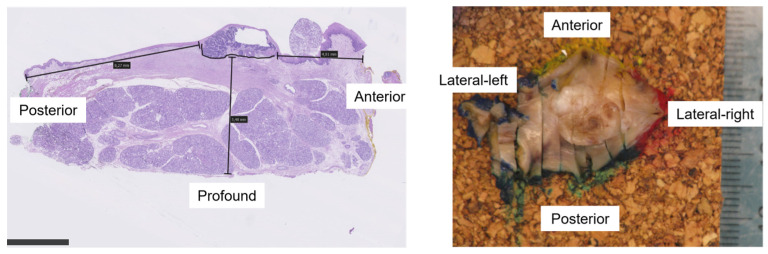
Schematic representation of the margin measurement direction as Anterior: towards the apex of the tongue; Posterior: towards the base of the tongue; Lateral-right: towards the floor of the mouth; Lateral-left: towards the back of the tongue; and Profound: depth.

A standard examination of the surgical specimen will be performed in the operation theater by the surgeon in order to assess the margins after resection. This usually includes inspection and palpation of the surgical specimen to assess if there are close/positive margins. Additionally, the surgeon will evaluate the margins at five specific areas (anterior, posterior, medial, lateral, and profound) and estimate the closest distance in mm from the tumor to the resection (free margin) for each area. See Figure 1 for the schematic of the definition of the margin measurement directions.

Then, the surgeon will attach the resected specimen to a piece of cork using pins and will mark the directions.

#### 3.2.2. 3D Ultrasound Scan (3Sonics) of the Surgical Specimen on-Site

**Figure 2:**
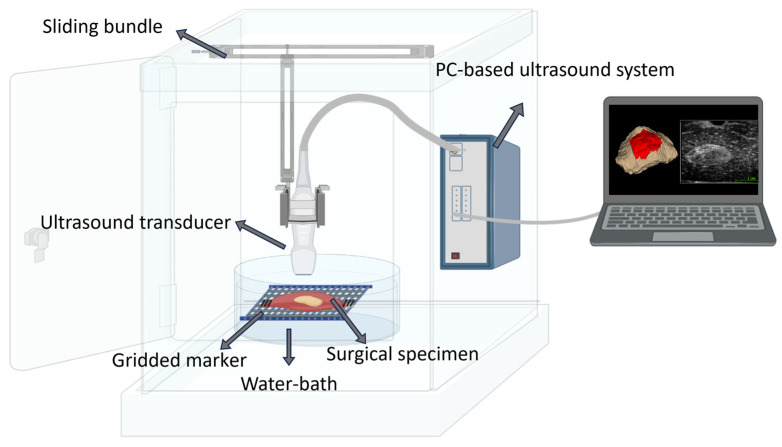
Schematic of components of the 3D ultrasound imaging method. The setup contains a PC-based ultrasound device, a mechanical arm, a gridded marker for volume calibration, a computer, and a water bath (Figure created with https://www.biorender.com/ (accessed on 30 May 2023)).

In the operation theater, the surgical specimen will undergo a 3D ultrasound scan. The 3D ultrasound scan will be performed using a portable custom-made setup, which we have named “3Sonics”. See Figure 2 for the different components of the 3D ultrasound imaging solution.

The 3D ultrasound imaging setup includes a slider (motorized mechanical arm) to move the transducer at a constant speed. A custom-made probe holder is connected to the slider to attach the ultrasound transducer and adjust the distance of the ultrasound probe front-face to the specimen in the depth direction. A water bath is designed to submerge the surgical specimen in saline water. We will pin a small plastic marker onto the cork next to the specimen for calibration and retrieval of the coordinate system. We have made a customized program in MATLAB (www.mathworks.com (accessed on 5 July 2023)) to generate 3D ultrasound volumes of the ex vivo surgical specimens. We will use an ArtUs EXT-1H ultrasound system (TELEMED, UAB Savanoriu, Lithuania) with a linear probe (L18-7H30-A5), which is ideal for near-field imaging and which provides detailed images of superficial structures. This probe has 192 elements, a 7–18 MHz frequency range, and a 30 mm field of view. The resolution in the probe sweep direction is 0.06 mm. Based on the inclusion criteria, including tumors with T1–T3 staging, we do not expect to scan depths greater than 4–5 cm, which will allow for high-resolution ultrasound scans. The ultrasound image optimization will be performed at the center position of the specimen prior to the mechanical sweep of the probe. The image optimization will mainly include the depth, frequency, gain, and focus adjustment to ensure the minimum required depth, the highest possible resolution and contrast, and the focus point at approximately the center of the tumor region. To minimize the artifacts, we will ensure that the ultrasound probe is correctly positioned and oriented. The total scanning time with setup adjustment and data acquisition has been estimated in pilot tests to be less than 10 min. The volume construction and calibration of the ultrasound data will be performed using the custom-made script in the commercially available programming tool MATLAB 2022b (www.mathworks.com (accessed on 5 July 2023)). 

Following the surgery, two experienced head and neck surgeons will manually segment the tumor region on the 3D ultrasound images. This process will involve identifying and delineating the tumor boundaries based on the echogenicity differences between the tumor and the surrounding healthy tissue. Segmentation will be conducted using the ITK-SNAP segmentation software (www.itksnap.org (accessed 5 on July 2023)) [48], a widely used and validated tool for medical image segmentation. The surgeons will measure the smallest margin (anterior, posterior, lateral, medial, and deep margin). The segmentation process will be performed on approximately 10–15 equally distanced planes throughout the tumor volume. These planes will be selected to provide a comprehensive representation of the tumor and its spatial relationship with the surrounding tissue. The segmented regions from these planes will then be interpolated (using the interpolation tool in ITK-SNAP) to construct a 3D representation of the entire tumor structure. This approach allows for a detailed visualization and measurement of the tumor and its margins from multiple perspectives.

In addition to the overall tumor segmentation, the surgeons will measure the deep margin at every segmented plane. This step will provide a detailed plane-by-plane assessment of the deep margin, which is crucial for determining the adequacy of the resection. The measurements from the 3D ultrasound will then be correlated with the histopathology results to evaluate the accuracy of the ultrasound-based measurements.

#### 3.2.3. MRI Scan of the Surgical Specimen in the Radiology Department

Immediately after the tumor resection, clinical examination by the surgeon, and the 3D ultrasound scan at the operation theater, the ex vivo surgical specimen will be transferred to the radiology department to allow for the acquisition of an MRI scan. The specimen will be oriented to maintain the same orientation as the 3D ultrasound scan. The surgical specimen pinned to the cork will be enclosed in a plastic box to ensure a stable positioning of the specimen and to minimize the possible pollution at the MRI setup.

A 1.5T MRI scan (Artist, GE Healthcare) will be performed using a wrist radiofrequency (RF) coil and a scan protocol with T2-weighted 2D MRI (1.0 mm slice thickness). The total examination time will be approximately 15 min. Two radiologists consultants will assess the surgical margins and the tumor region on the MRI scans over two rounds, similar to the procedure explained at Section 3.2.2.

#### 3.2.4. Tissue Preparation and Histopathology

After conducting the MRI scan in the radiology department, the surgical specimen will be returned to the pathology department for formalin fixation. 

In the pathology department, the samples will be treated using standard procedures to create formalin-fixed, paraffin-embedded (FFPE) blocks of tissue. The samples are first formalin-fixed with 10% formaldehyde for 24 h. Before starting the slicing procedure of the specimen by the pathologist, the formalin-fixated specimen will again undergo 3D ultrasound. Then a pathologist will perform parallel slicing, with almost equal intervals (~2 mm). The slices are then transferred to cassettes for further processing and embedded in paraffin to form FFPE blocks, which are cut at 4 μm and mounted on glass slides. Afterwards, the slides will be stained with hematoxylin and eosin (H&E) to visualize the tissue under the microscope. Two pathologists will assess the surgical margin status and will delineate the tumor on the slides. The tumor volume in milliliters (mL) will be estimated by the multiplication of the tumor area per slice and the cutting intervals. The pathologist will also measure the smallest margin at the posterior, anterior, lateral, medial, and profound directions (as described in Figure 1). The average measurement obtained by the two pathologists will be considered as the gold standard.

## 4. Clinical Outcome Definition

We will compare the surgical margin assessment in tongue cancer specimens by comparing three index tests (clinical examination, 3D ultrasound, and MRI) with the post-surgical histopathology assessments as the reference standard. 

The primary outcome will be reported as:

- The perioperative measurement of resection margins (mm) at five directions (Figure 1) with clinical exam and imaging compared to the post-surgical histopathology results (reference standard).
- The image-by-image comparison of the depth of invasion measurement (mm) from 3D ultrasound/MR imaging and histopathology slides.
- The depth of invasion (mm) comparison between in vivo and ex vivo ultrasound.
- The number of margins correctly classified as free (>5 mm), close (1–5 mm), or positive (<1 mm) margins by 3D ultrasound and MRI using histopathology findings as the reference.

The secondary outcome will be:

- The number of cases requiring adjuvant treatments (surgery or chemo/radiotherapy) due to T-site residuals.
- A change in tumor volume and resection margin measurements with 3D ultrasound imaging before and after formalin fixation.
- The time usage (minutes) and cost estimation for perioperative 3D ultrasound and MRI.

## 5. Statistics

There will be three index tests used for clinical examination, 3D ultrasound imaging, and MRI of the ex vivo specimen. The reference standard will be the final histopathology result. Each case will undergo five margin measurements (anterior towards the apex of the tongue, posterior towards the base of the tongue, medial, lateral, and profound/deep margins) by three index tests and one standard reference. Tumor and specimen volume will be calculated for each case. The deep margin will be measured at equally distanced planes of imaging in every case, and the results will be correlated to the corresponding deep margin measurements from the reference standard.

The participants will be excluded from the final analysis in case of any technical issues such as non-feasibility to perform parallel slicing of the surgical specimen or patients with MRI contradictions (pacemaker, metal implants not compatible with MRI, etc.). The number of excluded participants will be reported as non-conclusive cases and will be excluded from the study.

Margin measurements (quantitative data in mm) and tumor and specimen volume measurements (quantitative data in ml) will be presented as an averaged value over two measurement repetitions, as described in Section 3.2. The measurement differences between the index tests and the reference standard will be compared by a paired *t*-test. The degree of correlation will be expressed using Pearson’s correlation coefficient, in which the index tests and the reference standard will be considered to be significantly correlated when *p* < 0.05. To evaluate the agreement between the index tests and the reference standard, we will also analyze the data using Bland–Altman plots, which show the mean difference between the two techniques with a 95% confidence interval. This allows for easy interpretation of whether the differences between the index tests and the reference standard are clinically relevant. Correlation between the plane-by-plane deep margin measurements obtained by the reference standard and the index tests will be evaluated by *t*-test, and the threshold for significance correlation will be *p* < 0.05. The correlation between the depth of invasion measurements (mm) from in vivo ultrasound and the ex vivo ultrasound will also be reported.

The intra- and interobserver variability will be quantified by calculating the intraclass correlation coefficient (ICC). The ICC measures the reliability of the measurements between the two observers and is the measure of the degree of agreement among the observers in their assessment of margin status.

We will also classify the data into three categories of positive (<1 mm), close (between 1–5 mm), and free (>5 mm) margins. For each included case, we will compare the accuracy of each index test by computing the proportion of correctly classified margins with respect to the reference standard class. We will use the McNemar test to determine if the proportions of categories in the index tests differ significantly from those of the reference standard (paired categorical data). The results will be considered statistically significant when *p* < 0.05, with a 95% confidence interval. 

The mean time spent on delineation by the operators will be recorded and reported in minutes. Data will be analyzed with commercially available software (RStudio Team-2023). RStudio: Integrated Development for R. RStudio, Inc., Boston, MA, USA.

## 6. Power Calculation and Inclusion Period

A power calculation was based on the expected change in frequency between the free margin status when diagnosed by ultrasound imaging of the surgical specimen and when evaluated using the conventional procedure in the operation theater, without using ultrasound. The sample size needed to compare two independent binomial proportions is calculated to achieve a significance level of 0.05 and 90% power. The power calculation is based on results from Koning et al. [18] that found the frequency of free margin status to be 55% using ultrasound and 16% in the conventional cohort. A 2-sample paired binary sample size calculation results in the sample size of n = 27. By estimating a 10% drop-out rate, due to technical reasons, we will aim to include 30 cases. 

In Denmark, oral cavity cancer is the second most common head and neck cancer, and it has previously been reported with an incidence of 3.5 pr. 100,000. The uptake for the Rigshospitalet Center averages more than 90 cases annually, with 1/3 being tongue tumor cases [2,49]. In Denmark, all cancer investigations and treatments take place in public hospitals, free of charge, and financed by taxes. Based on the Copenhagen oral cavity squamous cell carcinoma database [6], we expect more than 30 patients annually to be diagnosed with tongue SCC, and these are the potentially eligible participants. Some of these patients will not fulfill the inclusion criteria, and some will decline to participate in the study. Therefore, we expect to include roughly 60% of the patients over a 1.5 year period. All enrolled patients are included in the analysis, with an intention-to-treat assumption [50]. We will register the age, sex, and final pathology staging of all the eligible participants to assess the comparability of the indexed population to the recurrent general tongue cancer population.

## 7. Ethics and Data Management

Ethical approval was granted in the form of an exemption letter from the Committee on Biomedical Research Ethics of the Capital Region of Denmark (registration number: H 22058519). Verbal and written informed consent will be obtained from every patient involved. All data will be stored on a Research Electronic Data Capture (REDCap) Database, and documentation requirements and data permission were approved by The Capital Region of Denmark (registration number: P-2021-319).

Trial registration: clinicaltrials (NCT05843032).

## 8. Discussion

Earlier studies have demonstrated the advantages of 2D ultrasound-guided tongue SCC resections [7,18,51]. This trial aims to explore whether a novel 3D ultrasound imaging technique of the tongue tumor offers a diagnostic rate higher than that obtained using MRI and clinical examination by the surgeon. If so, implementation of surgical margin assessment using 3D ultrasound may increase the free margin rate in tongue cancer surgery. This will improve the treatment of tongue tumor patients and will reduce healthcare costs. 

## Figures and Tables

**Table 1:** Overview of 3D ultrasound techniques.

Tracking Technique	Year	Reference	Aim	Highlights of the Technique
Electromagnetic sensor	2021	Bekedam et al. [21]	Intraoperative tongue tumor margin assessment	- Cost-effective - Convenient for localization - Magnetic field susceptible to metallic materials
	2020	Ruijter et al. [22]	3D geometry assessment of carotid artery	
	2017	Pelz et al. [23]	Direct visualization of internal carotid artery stenosis	
	2013	Ying et al. [24]	Cervical lymph node volume measurement	
Mechanical arm	2022	Sabiniok et al. [25]	Breast phantom study	- Sweep along a predefined direction - Volume reconstruction based on the position and orientation of the probe - Image quality degradation in directions other than the probe sweep path
	2012	Yan et al. [26]	Needle tracking in prostate brachytherapy	
Optikal tracker	2016	Cenni et al. [27]	Phantom study	- Direct line of sight between the camera and trackers required during the scan [28]
3D probe	2019	Chung et al. [29]	Tonsillar volume measurement	- Lower quality of the image using 3D probes compared to 2D probes - Expensive and bulky - Limited field of view
	2022	Makouei et al. [30]	Animal model	
	2014	Zhao et al. [31]	Acquiring and analyzing 3D ultrasound images of deep vein thrombosis	
Sensorless	2006	Housden et al. [32]	Animal model	- Lower accuracy of volume reconstruction [20]
	2002	Li et al. [33]	Simulation study	

**Table 2:** Examples of imaging modalities used for margin assessment.

Imaging Technique	Year	Number of Cases	Reference	Diagnostic Conclusion
The use of 2D ultrasound and MRI	2019	83	de Koning et al. [41]	For preoperative tumor staging in oral cancer, the tumor thickness is better estimated by the use of ultrasound compared to MRI.
The use of 2D ultrasound and MRI	2011	65	Lodder et al. [42]	Tumor thickness in oral cancer is an important predictive marker for lymph node metastases.
MRI and clinical examination	2016	53	Alsaffar et al. [43]	There is a high correlation between pathological, radiological, and clinical examinations in the measurement of tongue tumor thickness in deep tumors (≥5 mm).
Time-resolved fluorescence spectroscopy	2019	4	Gorpas et al. [44]	Label-free and real-time assessment and visualization of tissue biochemical features during oral tumor robotic surgery procedures have the potential to improve intraoperative decision making during transoral robotic surgery.
CT	2019	4	Kahng et al. [45]	Intraoperative imaging improves localization accuracy when targeting submucosal beads in cadaver heads during operative laryngoscopy.
The use of 2D ultrasound	2021	10	De Koning et al. [7]	The use of ultrasound-guided tongue SCC is feasible and improves margin control.
Fluorescence	2018	21	Gao et al. [46]	Fluorescence can be used as a sensitive method for guiding surgery in head and neck cancers, increasing the probability of complete resections and improving oncologic outcomes.

**Table 3:** Eligibility criteria for enrollment of the study participants (inclusion and exclusion).

Criteria	Description
Inclusion	Patients with biopsy-proven oral tongue squamous cell carcinoma scheduled for surgical treatment. T1–T3 staging on cross-sectional imaging.
Exclusion	Age < 18 years. T4 staging. Unable to understand the verbal or written information. Prior radiotherapy treatment of oral cavity cancer.

## Data Availability

Not Applicable.
